# Anti-apoptotic mechanisms of HIV: lessons and novel approaches to curing HIV

**DOI:** 10.1007/s00018-012-1239-3

**Published:** 2012-12-30

**Authors:** Nathan W. Cummins, Andrew D. Badley

**Affiliations:** Division of Infectious Diseases, Mayo Clinic, 200 1st Street SW, Rochester, MN 55905 USA

**Keywords:** Human immunodeficiency virus, Latency, Apoptosis, Immune evasion

## Abstract

Past efforts at curing infection with the human immunodeficiency virus (HIV) have been blocked by the resistance of some infected cells to viral cytopathic effects and the associated development of a latent viral reservoir. Furthermore, current efforts to clear the viral reservoir by means of reactivating latent virus are hampered by the lack of cell death in the newly productively infected cells. The purpose of this review is to describe the many anti-apoptotic mechanisms of HIV, as well as the current limitations in the field. Only by understanding how infected cells avoid HIV-induced cell death can an effective strategy to kill infected cells be developed.

## Introduction

The loss of CD4 T cells during acute and chronic HIV infection occurs predominately through aberrant induction of cell death, including through apoptosis. Apoptosis is one type of a growing number of related mechanisms for programmed cell death [1]. It is characterized phenotypically by cell membrane blebbing, cellular shrinking, and nuclear and DNA fragmentation. Apoptosis has classically been categorized as occurring via one of two main pathways: (1) the extrinsic pathway, induced by a death ligand (tumor necrosis factor [TNF], Fas ligand, or TNF-related apoptosis inducing ligand [TRAIL] engagement of a death receptor; or (2) the intrinsic pathway, induced by a number of intracellular signals, including oxidative stress, genotoxic stress, UV irradiation, and growth factor withdrawal. The signaling pathways that ultimately lead to apoptosis are highly regulated by overlapping mechanisms, and include a number of pro-apoptotic proteins (e.g., Bim, Bid, Bad, Bax, Bak, and PUMA, etc.) as well as anti-apoptotic proteins (e.g., Bcl-2, Bcl-XL, XIAP, and FLIP, etc.). Some forms of apoptosis are dependent on depolarization of the mitochondrial outer membrane with release of mitochondrial contents (e.g., cytochrome C, SMAC) that promulgate the apoptotic signal, whereas other forms are mitochondria-independent. In addition, some but not all forms of apoptosis are dependent on the activity of a family of proteins called caspases (cysteine-dependent aspartate-directed proteases), which can act either as regulators or effectors of apoptosis.

Many controversies remain in the understanding of HIV-induced apoptosis. For instance, does apoptosis occur predominately in infected or uninfected, so called bystander, cells? Which of the many pro-apoptotic mechanisms proposed to account for CD4 T cell losses over time predominate in clinical HIV disease? And most importantly, why does HIV induce apoptosis in the host target cells? Nearly 20 years of research in HIV-induced apoptosis has largely failed to find definitive answers to these fundamental questions.

We refer the reader to a number of recent reviews that summarize the latest understanding of the hypothesized pro-apoptotic mechanisms and effects of HIV, a discussion of which is beyond the scope of this manuscript [2, 3]. However, it is increasingly clear that the predominate reason HIV induces apoptosis in the host target cell is, paradoxically, to ensure viral replication. In fact, HIV replication is (1) increased in immortalized T cell lines induced to express pro-apoptotic proteins (FasL, FADD [Fas-associated death domain protein], and p53); (2) decreased in cells overexpressing anti-apoptotic proteins (Bcl-2, FLIP [FLICE-inhibitory protein], Bcl-XL, and XIAP [X-linked inhibitor of apoptosis protein]); (3) decreased in cells with knockdown of pro-apoptotic proteins (Bax and FADD); and (4) decreased in the setting of inhibition of caspase 3 activity [4–6]. Importantly, treatment with the HIV envelope protein Gp120 or expression of Casp8p41 (a unique cleavage fragment of procaspase 8 generated by HIV protease), both of which are associated with HIV-induced apoptosis, increases NF-κB-dependent HIV-LTR (long terminal repeat) transcription compared to untreated cells or cells treated with control proteins [7, 8]. This suggests that the critical link between HIV-induced apoptosis and replication is in activation of NF-κB, through a caspase 8-dependent mechanism, a survival mechanism that has been co-opted by a number of other viruses [9].

The purpose of this review is to summarize the current understanding of another fundamental question regarding HIV-induced apoptosis: How does HIV prevent death in the infected host cell long enough to promote viral replication and spread and establishment of the latent viral reservoir? This question is of paramount importance. If one could determine how HIV prevents premature infected cell death, then inhibiting this effect could lead towards a viral eradication strategy aimed at killing all infected cells, and eventual cure of the infection.

## Grains of salt

Studying HIV-induced apoptosis has several notable limitations to which the readers’ attention must be drawn, as inferences made on experimental data are inherently difficult. First, phenotypic and molecular definitions of apoptosis have evolved significantly over time, and there is increasing recognition of overlap between alternate mechanisms of cell death, including necrosis, necroptosis, paraptosis, autophagy, and others [10, 11]. Second, until recently, small animal models of HIV are limited in pathologic relevance, and non-human primate models are both financially and temporally expensive. Furthermore, access to relevant human samples is often limited by invasiveness and patient discomfort. Therefore, much of the experimental data relies on in vitro or ex vivo cellular or tissue models, which necessarily do not mimic the immunologic complexity of an HIV-infected person. Third, these models often rely on exogenous administration, or forced overexpression of HIV-associated proteins; however, neither the biologically relevant intracellular nor tissue concentrations of the HIV-associated proteins are agreed upon. Other investigators infect cells with single-deletion, replication-deficient mutants, making the results of these models of questionable physiologic relevance. Finally, measurement of markers of apoptosis in concert with meaningful markers of HIV infection cannot reliably differentiate between apoptotic-and-infected or apoptotic-and-uninfected cell deaths. This is because cells undergoing apoptosis activate proteases and nucleases that degrade cellular targets of laboratory methods to detect infection, i.e., antibodies to detect HIV-associated proteins, and complementary nucleic acids to detect HIV RNA or cDNA.

## Lessons from viral kinetics

In vitro models of HIV infection indicate (and mathematical models of viral decay in HIV-infected patients who initiate antiretroviral therapy confirm) that the single round life cycle of HIV in productively infected cells is 2–3 days [12–14]. This suggests that in productively infected cells, HIV-associated antiapoptotic mechanisms need not be particularly robust or long-lasting. Furthermore, individual CD4 T cells are generally infected by a single HIV virion, and upon cell death release 25–200 progeny virions per cell [14]. This would suggest that HIV proteins present early in the infection cycle, which are derived solely from the single infecting virion, would exist in relatively low concentrations. However, after transcription of viral mRNA and translation of viral proteins in preparation for virus assembly and release, the same proteins would be expressed at a relatively much higher intracellular concentration. As will be discussed below, this may provide a framework upon which to interpret seemingly contradictory results obtained in studies describing both pro- and anti-apoptotic effects of individual HIV-associated proteins.

## Early events in the HIV virus life cycle

Binding of the HIV envelope protein Gp120 to the host cell CD4 receptor induces a conformational change in the Gp120, allowing binding to the cellular co-receptor, principally CCR5 or CXCR4. This attachment is followed by fusion of the viral and host membranes mediated by Gp41, and release of viral genetic material and a number of virus-associated proteins into the cytoplasm of the cell. The viral proteins released during uncoating of the virus include reverse transcriptase, structural proteins, and the viral accessory proteins Nef, Vif, and Vpr. Since Gp120 ligation of the CD4 receptor and co-receptors induces pro-apoptotic signals through a number of pathways [2], and has been implicated in uninfected bystander T cell apoptosis, it is likely that these early viral-associated proteins protect the newly infected cell from premature cell death.

For instance, Vpr is an HIV-accessory protein present in high copy number in mature virions, and is released into the cytoplasm upon uncoating of the infecting virus; it has been variously described to have both pro- and anti-apoptotic properties based on the infection model and protein concentration [2]. Jurkat T cells stably expressing low levels of Vpr are resistant to both intrinsic and extrinsic apoptotic signals compared to control cells, and this is associated with increased Bcl-2 and decreased Bax expression [15]. Jurkat cells infected with HIV have a transient resistance to apoptosis induced by tumor necrosis factor (TNF) and cyclohexamide at 48 h after infection, which reverses at 72 h [16]. This apoptotic-resistant phenotype of HIV infection is dependent on Vpr expression, and occurs predominately in productively infected cells as opposed to bystander cells [16]. However, in later stages of infection, the opposite effect is seen, in that apoptosis in the absence of additional stimuli is increased in productively infected Jurkat cells compared to uninfected cells. Furthermore, inhibition of Vpr using antisense oligodeoxynucleotides reverses this increased spontaneous apoptosis [16]. Similar results are demonstrated in HIV-infected primary cells. These experiments show that Vpr expressed in low levels early in infection are anti-apoptotic, but in higher concentrations later in infection the same protein is pro-apoptotic.

Vpr also induces G2 cell cycle arrest in infected cells, one effect that has been implicated in HIV-induced apoptosis. However, early in infection, the HIV-accessory protein Vif induces proteosomal degradation of Vpr, similar to Vif’s function of inducing degradation of the ABOBEC3G host restriction factor. Therefore, expression of Vif early in the infection cycle may delay Vpr-induced G2 cell cycle arrest, allowing for early infected cell survival [17].

Nef is a polyfunctional HIV-associated protein that downregulates the expression of a number of cell surface receptors on the infected cell, including MHC Class I molecules and CD28. In addition, Nef expression, and to a lesser extent Vpu and Gp120, in HIV-infected cells decreases CD4 surface expression, decreasing the likelihood of superinfection with a second virus [18]. Superinfection increases infected cell apoptosis [18]; therefore, this may represent another antiapoptotic mechanism early in the viral life cycle. Nef also directly binds to p53 through its N-terminal end and decreases the intracellular half life of p53 and p53-dependent transcriptional activation in in vitro HIV infection [19]. As additional evidence of Nef’s anti-apoptotic effects, endogenous expression of Nef in MOLT-4 cells protects against p53-dependent UV-induced apoptosis. Since p53 signaling has been implicated in apoptosis induced by the HIV Gp120 [20], it is possible that early expression of Nef inhibits pro-apoptotic signals induced by viral attachment and entry.

A number of cellular proteins, in addition to the viral proteins discussed above, are packaged into and accompany infecting HIV virions [21], and could have an early anti-apoptotic effect. For instance, the cellular protein kinase mitogen-activated protein kinase 1 (MAPK1), also known as extracellular signal-regulated kinase 2 (ERK2), is incorporated into the virion, and has been shown to be necessary for nuclear translocation of the HIV pre-integration complex [22]. Phosphorylation by MAPK1/ERK2 regulates the activity of a number of apoptotic regulatory proteins, and inhibition of MAPK1/ERK2 activity inhibits cellular proliferation and decreases survival [23]. It is possible that MAPK1/ERK2 incorporated into HIV virions could have a similar function in early stages of cellular infection with the virus, although this has not been directly studied. Additional examples of cellular proteins that are incorporated into HIV that have anti-apoptotic properties include heat shock protein 70 (Hsp70) and cyclophilin A [21].

A recent study published in *Cell* by Doitsh and colleagues has caused a true paradigm shift in the understanding of HIV-induced apoptosis, and so-called “bystander” apoptosis [24]. Using a model of ex vivo infection of human lymphoid tissue, they demonstrated that incomplete reverse transcription of the viral genetic material results in the accumulation of cytotoxic DNA material, resulting in paraptosis (a form of apoptosis) of cells nonpermissive of viral replication. Therefore, bystander cells, instead of being truly uninfected, may be non-productively infected, and this condition of non-productive infection drives cell death. It is possible, then, that the ultimate fate of the early infected cell is dependent upon mechanisms that promote survival in the face of this cytotoxic insult, and may rely on the above-mentioned anti-apoptotic viral or cellular proteins that accompany the infecting virion.

## Latency

After reverse transcription of the viral genome and integration of the cDNA into the host genome, an important minority of cells enters a period of latent infection, defined by the absence of transcription of the viral message and production of viral proteins. This is in contrast to chronic infection, in which infected cells produce and release progeny virions, but do not succumb to HIV-induced cell death. The molecular determinants of HIV latency, including histone deacetylases [25] as targets for non-toxic viral activation, are the “Holy Grail” of current research efforts at novel viral eradication strategies. X-linked inhibitor of apoptosis protein (XIAP), a member of the IAP family, inhibits apoptosis by directly inhibiting caspase 3 and caspase 9 activity, as well as activating JNK signaling [26]. One study suggests that XIAP expression is increased in latently HIV-infected T cells compared to uninfected cells, and chemical inhibition of XIAP activity sensitizes the latently infected cells to apoptosis [27]. Similarly, we have observed decreased expression of procaspase 8 in central memory CD4 T cells, which serve as an in vivo latent viral reservoir (unpublished observations). However, more research is needed in this area, as current in vitro models of latent HIV infection rely heavily on immortalized laboratory cell lines with integrated HIV virus, the relevance of which to in vivo viral latency is unclear.

Similarly, the chronically HIV-infected H9 T cell line exhibits a significantly altered apoptotic gene expression profile by microarray analysis compared to the uninfected parent cell line [5]. Infected H9 cells have increased expression of nine antiapoptotic and seven proapoptotic genes, and decreased expression of six antiapoptotic and 12 proapoptotic genes, possibly contributing to cell survival in the setting of chronic infection. Chronically HIV-infected HUT78 T cells express less DAP kinase, p19ARF, p53, and p21WAF1, all proteins associated with death-receptor signaling, compared to uninfected control cells [28]. This is associated with increased resistance to FasL-mediated apoptosis in infected cells compared to control.

## Late events in the HIV virus life cycle

A number of cellular activation signals and transcription factors, including NF-κB, can initiate the transcription of integrated viral message to begin the viral replication processes. One of the first viral proteins produced following reactivation is Tat (transactivator of transcription), which further enhances transcription of viral genes by binding to the transactivation response element within the HIV-LTR. Endogenous expression of Tat in Jurkat T cells decreases apoptosis induced by TNF, FasL, TRAIL, and T cell receptor ligation compared to control cells [29, 30]. Furthermore, Jurkat T cells transfected with a Tat-expression vector are less susceptible to apoptosis induced by subsequent infection with HIV compared to mock transfected cells. A number of mechanisms have been proposed to account for Tat’s anti-apoptotic effects. Endogenous expression of Tat in HeLa, Jurkat T cells, and PBMCs results in increased Bcl-2 expression compared to control cells [31, 32]. This is likely a direct transcriptional activation, as the C-terminal end of Tat binds to two regions in the Bcl-2 promoter, whereas the N-terminal end of Tat is required for HIV-LTR transactivation [32]. Also, picomolar concentrations of exogenous Tat are sufficient to increase Bcl-2 expression in treated cells compared to untreated cells [31]. One study indicated that endogenous expression of Tat in two immortalized cell lines of non-lymphoid origin (COS and H1299) is associated with increased ubiquitination and degradation of Tip60, a pro-apoptotic protein involved in the DNA damage response [33]. This confers resistance to apoptosis induced by actinomycin D in these cells. Jurkat T cells infected with HIV for 14 days express less Tip60 and are more resistant to actinomycin D-induced apoptosis compared to uninfected cells. However, the in vivo relevance of this potential anti-apoptotic mechanism is not known.

c-FLIP (FLICE-inhibitory protein) is a cellular regulator of apoptosis that inhibits caspase 8 and 10 activation at the DISC. Endogenous expression of HIV-1 Tat in Jurkat T cells results in increased c-FLIP expression and a TRAIL-resistant phenotype compared to control cells [34]. c-FLIP expression was not different between PBLs isolated from HIV-infected and HIV-uninfected subjects in one small study [35]. However, c-FLIP expression was found to be significantly increased in PBLs from slow- or non-progressive HIV-infected subjects compared to chronic progressive HIV-infected subjects [36].

As mentioned above, Vpr expression induces G(2)/M cell cycle arrest. Coincident with this cell cycle arrest, Vpr also induces expression of survivin in HIV-infected T cells [37]. Survivin is another member of the IAP family, which inhibits apoptosis by binding to XIAP and increasing XIAP’s stability against proteosomal degradation, and by sequestering SMAC/Diablo from interacting with XIAP [38]. Induction of survivin expression by Vpr may then delay HIV-induced mitochondrial dependent apoptosis. This effect may be cell-type dependent, since Vpr expression in monocytes downregulates the expression of another member of the IAP family, c-IAP1, possibly contributing to Vpr’s pro-apoptotic effects [39].

Reactive oxygen species have been implicated in HIV-induced apoptosis. The HIV envelope gene also encodes for a viral homologue of the human glutathione peroxidase (GPX) [40]. HIV GPX-transfected cells are resistant to apoptosis induced by exogenous ROS. Interestingly, HIV GPX sequence analysis from infected patients revealed that HIV long-term non-progressors had a decreased frequency of loss of function mutations in the GPX gene compared to patients who had progressed to AIDS, suggesting an in vivo antiapoptotic effect of functional GPX expression.

Bad (bcl-associated death promoter protein) is a cellular protein that in a dephosphorylated state binds to anti-apoptotic proteins, allowing unopposed Bax/Bak dependent mitochondrial depolarization and resulting apoptosis. Infection of Jurkat T cells with an HIV pseudovirus containing wild-type Nef results in significantly less apoptosis over time than infection with a pseudovirus containing a mutant Nef [41]. This anti-apoptotic effect of Nef was associated with p21-activated kinase-dependent phosphorylation and inactivation of Bad.

## Differential effects in myeloid cells

Myeloid lineage cells, including monocytes and macrophages, by virtue of expression CD4 and the HIV-coreceptor CCR5, are permissive of HIV infection, but are largely spared the cytopathic effect associated with HIV replication, and therefore likely contribute to the long-lived viral reservoirs that impede viral eradication. This suggests that selective impairment of apoptosis in myeloid cells must occur by mechanisms unique to those cells. Evidence for this, though, is mixed, and varies based on what experimental conditions are used.

Early experiments with the promonocytic U937 cell line, and the myeloblastic PLB-985 cell line, when chronically infected with HIV, demonstrated that resistance of these cells to HIV-induced apoptosis was associated with constitutive NF-κB activity in infected cells compared to uninfected cells. However, HIV-infected cells had an increased susceptibility to apoptosis induced by additional stimuli, such as TNF and the protein synthesis inhibitor cycloheximide [42]. In contrast, chronically HIV-infected U937 cells are more resistant to apoptosis induced by DNA-damaging agents compared to uninfected cells or latently infected U937 cells [43]. However, activation of viral replication in the latently infected macrophages renders these cells also resistant to apoptosis induced by DNA-damaging agents, suggesting that viral proteins expressed in these cells may have predominately anti-apoptotic effects. This is important because current research aimed at eradication of latent viral reservoirs is predicated on the hypothesis that activating viral replication will increase susceptibility to apoptosis-inducing agents. This may be a sound strategy for latently infected T cells, but this may not be the case in macrophages. In addition, chronically HIV-infected U937 cells are more resistant to apoptosis induced by hydrogen peroxide or the protein kinase inhibitor staurosporine compared to uninfected cells [44]. Induction of increased viral replication in these cells with TNF or PMA did not alter susceptibility to these apoptosis-inducers.

Chronically HIV-infected U937 cells exhibit a substantially different apoptosis-related gene expression profile compared to chronically infected CD4 T cells lines (H9 and MT4), including differential expression of NME3, STK17A, CD74, and HIPK3 [5]. Similarly, circulating monocytes from HIV-infected donors exhibit differential expression of 38 apoptosis-related genes compared to uninfected donors [45]. These genes, linked to p53, CD40L, TNF, and MAPK signaling pathways, are associated with increased resistance to FasL-induced apoptosis in monocytes from HIV-infected donors compared to uninfected donors.

In vitro HIV infection of cultured monocyte-derived macrophages (MDMs) results in production of TNF and NF-κB activation, and yet the cells do not die [46]. This resistance to apoptosis is associated with increased expression of Bcl2 and Bcl-XL, and decreased Bax expression. Since the increased expression of Bcl2 and Bcl-XL is not fully reversed by an NF-κB inhibitor, it is likely that HIV-associated proteins modulate the expression of these proteins in macrophages as well in an NF-κB-independent manner. Another study in a similar model showed that in vitro HIV infection of MDMs also results in hyperphosphorylation of the pro-apoptotic protein Bad, resulting in its inactivation and consequent MDM resistance to HIV-induced apoptosis [47]. This effect was dependent on functional Nef expression, since infection of MDMs with a single deletion mutant pseudovirus lacking functional Nef resulted in significantly increased apoptosis compared to wild-type Nef [47]. On the other hand, in vitro HIV infection of MDMs also results in impaired NF-κB-dependent responses to TLR2 and TLR4 agonists at a transcriptional level compared to uninfected cells [48]. Since these infected macrophages do not die during in vitro infection, this suggests that selective inhibition of NF-κB in these cells may preserve infected cell viability.

## Immune evasion

In addition to avoiding HIV-induced infected cell death, it is hypothesized that anti-apoptotic effects of HIV infection may have evolved in order for the virus to evade the immune response to infection, either by evading detection of infected cells by innate censors, or by inducing resistance of infected cells to the various death ligands used by immune effector cells to kill infected target cells.

For instance, both cytotoxic T lymphocytes and NK cells are important components of the innate immune response to HIV. NK cells express a class of receptors called killer cell immunoglobulin-like receptors (KIR), which interact with MHC Class I ligands, and can be either inhibitory or activating in antiviral activity [49, 50]. Effective killing of HIV-infected CD4 T cells by autologous NK cells is dependent on the type and activity of KIR expression on the NK cell and the type of MHC Class I expression on the target cell [51]. On the host side, numerous studies demonstrate that polymorphisms in the KIR and HLA loci are associated with variations in HIV control, disease progression, and immunologic recovery on antiretroviral therapy [52–54]. Furthermore, HIV has evolved a number of ways to circumvent these protective responses. As mentioned above, the HIV Nef protein downregulates the expression of MHC class I molecules on the surface of infected cells, which provides for one mechanism of evasion of recognition by cytotoxic T lymphocytes and NK cells [55]. In addition, HIV sequence polymorphisms that enhance inhibitory KIR activity are selected under the immunologic pressure of KIR + NK cells in chronically HIV-infected individuals [56]. These HIV mutations are then associated with reduced antiviral activity of the NK cells, adding another potential mechanism of immune escape.

Regarding resistance to death ligands, multiple investigators have shown that stable expression of Nef in Jurkat T cells renders those cells less susceptible to Fas- and TNF-induced apoptosis compared to control cells [57–59]. One mechanism for this appears to be that Nef binds to ASK-1 (apoptosis signal-regulating kinase 1), preventing its dissociation from thioredoxin, a requisite step in Fas and TNF receptor-mediated activation of JNK1 [59]. Notably, this effect was also demonstrated in HIV-infected primary CD4 T cells. Also, coculture of HIV-infected primary CD4 T cells with autologous macrophages resulted in a decrease in apoptotic infected cells through hyperactivation of NF-κB by TNF stimulation, an effect that was not present in cells infected with a single deletion Nef-mutant virus [57].

TRAIL is another death ligand used by innate immune effector cells to clear virally infected cells [9]. Monocytes prestimulated with exogenous Tat are more resistant to apoptosis induced by recombinant TRAIL compared to unstimulated monocytes, an effect associated with increased Bcl-2 expression [60]. Other effects of individual HIV proteins on TRAIL sensitivity have been discussed above. HIV-infected macrophages are resistant to TRAIL-mediated apoptosis [61]. This has been associated with increased macrophage colony-stimulating factor (M-CSF), decreased TRAIL-R1 expression, and increased expression of the anti-apoptotic proteins Mcl-1 and Bfl-1 [61]. Furthermore, HIV-infected dendritic cells are resistant to TRAIL-dependent NK cell-mediated killing [62]. This resistance in dendritic cells to TRAIL-induced apoptosis is associated with increased expression of c-FLIP and c-IAP2 in infected dendritic cells.

TRAIL resistance in HIV-infected cells is not limited to myeloid cells. Primary CD4 T cells from HIV-infected subjects are resistant to TRAIL-induced apoptosis by autologous plasmacytoid dendritic cells, despite expression of functional TRAIL on the dendritic cells and TRAIL receptors on the CD4 T cells [63]. In fact, we have shown that HIV-infected CD4 T cells, despite expression of functional TRAIL and TRAIL receptors, do not undergo TRAIL-dependent killing, since immunodepletion with an anti-TRAIL antibody does not inhibit HIV-induced apoptosis in vitro. HIV infection induces alternative splicing of the TRAIL message, producing a novel TRAIL splice variant—TRAIL-short (TRAIL-s)—that binds selectively to TRAIL-R2 but does not induce apoptosis [64]. Knockdown of TRAILs in HIV-infected cells increases TRAIL sensitivity. Furthermore, TRAILs is detectable in serum and PBMCs of HIV-infected patients, suggesting that this anti-apoptotic mechanism may contribute to immune evasion of HIV-infected cells in vivo.

## Mutational escape of antiretroviral therapy

Inhibition of viral replication with antiretroviral therapy decreases HIV-induced apoptosis, and some antiretrovirals, particularly the protease inhibitors, have intrinsic anti-apoptotic activity independent of their antiviral effects [65]. On the other hand, mutational escape of suppressive antiretroviral therapy, which results in breakthrough viral replication, does not always result in increased CD4 T cell apoptosis, suggesting other viral anti-apoptotic mechanisms in these rare situations. For instance, certain resistance mutations, which develop in the gp41 subunit of Env during treatment with enfuvirtide, are associated with a decreased ability to induce fusogenic apoptosis in bystander cells compared to the wild-type virus [66]. Likewise, we have described resistance mutations in HIV protease that occur during treatment with protease inhibitors that impair the virus’ ability to produce an HIV-specific pro-apoptotic protein—Casp8p41—compared to wild-type protease, and thereby decrease apoptosis in infected cells [67]. While the evolutionary benefit of these two effects is unclear, it is possible that the impaired ability to induce apoptosis in these mutant viruses is a compensatory mechanism for the decreased replicative fitness induced by antiretroviral therapy.

## Toward an HIV cure

Since the description of the “Berlin patient”, who appears to have been functionally cured after two myeloablative peripheral blood stem cell transplants including one from a donor with a CCR5 deletion, and the recent description of two HIV-infected patients with undetectable HIV DNA after reduced intensity conditioning peripheral blood stem cell transplants, there has been a resurgence in the efforts to find a cure for chronic HIV infection [68, 69]. These efforts largely rely on various methods including gene therapy, immune-based therapies, and targeted reactivation of latent virus. The intent of the latter is that newly productively infected cells will die via one of the number of pro-apoptotic mechanisms implicated in HIV-induced cell death [70]. Unfortunately, however, accumulating evidence suggests that reactivation of latent viral reservoirs alone does not lead to infected cell death, and may actually increase viral burden [71, 72, 73]. This phenomenon is likely due to an HIV-induced anti-apoptotic state within the infected cell. Sensitization to pro-apoptotic stimuli, akin to chemotherapeutic combination treatment of apoptotic-resistant cancer cells, may be the solution to this problem. For instance, stimulation of latently infected cultured central memory T cells with αCD3/αCD28 antibodies leads to effective depletion of virally infected cells, whereas homeostatic signals, i.e., IL2 and IL7, leads to preservation of the infected cells [74]. An alternative strategy that has been explored in vitro is ex vivo antigenic stimulation of HIV-specific cytotoxic T lymphocytes to increase the CTL’s cytolytic effectiveness against reactivated cells [73].

## Conclusions

**Fig. 1:**
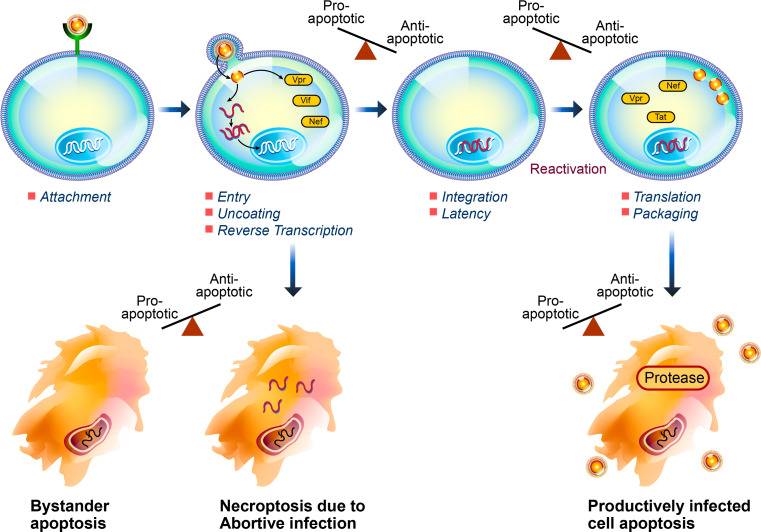
HIV infection induces cell death by a number of pro-apoptotic pathways, including bystander apoptosis, necroptosis induced by abortive infection, and apoptosis of productively infected cells. HIV-associated anti-apoptotic cellular effects, reviewed herein, serve to promote infected cell survival through the establishment of latency, reactivation, and viral replication, which is ultimately dependent on death of the infected cell

While many questions remain, all evidence suggests that HIV has evolved a number of mechanisms to interrupt apoptotic signaling and alter the apoptotic susceptibility of infected cells in order to delay premature cell death and evade the immune response with the result of promoting viral replication and persistence through the establishment of a latent viral reservoir (Table 1). The pleomorphic pro- or anti-apoptotic attributes of several of the HIV viral proteins could potentially be explained by the timing of expression and relative intracellular concentrations during the viral life cycle (Fig. 1). Establishing which of these mechanisms identified in the varying in vitro models occur in HIV-infected patients, and discovering additional novel anti-apoptotic mechanisms, will be most important in advancing the search for a safe and effective eradication strategy.

**Table 1:** A summary of anti-apoptotic mechanisms of HIV

Down-regulation of cell surface receptor expression
Alteration of intracellular protein expression levels, or the “apoptotic milieu”
Modulation of protein phosphorylation
Regulation of cell cycle progression
Evasion of innate and adaptive immune responses to infection
